# Potential Uses and Challenges of Three-dimensional Printing in Cardiothoracic Surgery in Africa - a Narrative Review

**DOI:** 10.21470/1678-9741-2024-0301

**Published:** 2025-09-11

**Authors:** Victory Bassey Effiom, Koustav Biswas, B.V. Sai Chandran, Sulayman el Mathari, Victor Femi-Lawal, Eric Vinck, Sameer A Hirji

**Affiliations:** 1 Department of Medicine and Surgery, Faculty of Clinical Sciences, University of Calabar, Calabar, Nigeria; 2 Department of Cardiothoracic & Vascular Surgery, Jawaharlal Institute of Postgraduate Medical Education and Research (JIPMER), Puducherry, India; 3 Department of Cardiothoracic Surgery, Amsterdam University Medical Center, Amsterdam, The Netherlands; 4 Department of Medicine and Surgery, College of Medicine, University of Ibadan, Ibadan, Nigeria; 5 Department of Cardiac Surgery, Colsubsidio Cardiovascular Clinic Calle 100, Bogota, Colombia; 6 Division of Cardiac Surgery, Brigham and Women’s Hospital, Harvard Medical School, Boston, Massachusetts, United States of America

**Keywords:** Three-Dimensional Printing, Cardiothoracic Diseases, Africa, Technology, Standard Care.

## Abstract

Three-dimensional (3D) printing is an innovative technology with increasing and emerging potential in cardiothoracic surgery. This technology has significantly impacted translational research, education, and clinical practice. In high-income countries, 3D printing has vastly broadened the understanding of the cardiovascular system and helped in surgical planning by facilitating the nuanced creation of patient-specific cardiac models with exact precision to allow the development of personalized devices and surgical tools to facilitate improved patient outcomes. However, in low-income countries, such as those in Africa, there is limited access to 3D printing technology. The growing burden of complex cardiovascular diseases in Africa warrants the need for this technology to improve the standard of care for cardiac surgical patients.

This review discusses the fundamentals of 3D printing, its relevance to current disease burdens in the context of the African population, its current state and future prospects in African cardiac care, its unmet needs, challenges, and how to implement it in the continent.

## INTRODUCTION

**Table:** 

Abbreviations, Acronyms & Symbols				
ASD	= Atrial septal defects		MRI	= Magnetic resonance imaging
CAD	= Coronary artery disease		SLA	= Stereolithography
CHD	= Congenital heart disease		SLS	= Selective laser sintering
CT	= Computed tomography		STL	= Standard Tessellation Language
3D	= Three-dimensional		TAVR	= Transcatheter aortic valve repair
DICOM	= Digital imaging and communications in medicine		TEE	= Transesophageal echocardiogram
DMLS	= Direct metal laser sintering		TMVR	= Transcatheter mitral valve repair
FDM	= Fused deposition modelling		UV	= Ultraviolet
FFR	= Fractional flow reserve		VDM	= Vascular deformity mapping
MCS	= Mock circulatory systems		VHD	= Valvular heart disease
MJF	= Multi Jet Fusion		VSD	= Ventricular septal defects
MR	= Magnetic resonance			

Three-dimensional (3D) printing is a fabrication technique that converts digital structures to physical models^[1]^. These digital structures are derived from imaging modalities such as magnetic resonance imaging (MRI), 3D ultrasound, and computed tomography (CT)^[2]^. In recent years, there has been an increase in 3D printing technology in the medical field. This trend has been seen in the creation of customized implants, surgical tools, prosthetics, fixtures, and patient-tailored 3D-printed models for surgical preparation^[3-9]^. These models are used in preoperative planning, surgical simulations, intraoperative guidance, patient education, and training of medical students and residents^[10-12]^. In cardiothoracic surgery, patient-specific 3D-printed models are currently mainly helpful in surgical procedures with complex anatomy such as congenital cardiac surgery^[13,14]^. The integration of 3D printing in cardiothoracic surgery has the potential to revolutionize patient care in Africa. The prevalence of congenital heart disease (CHD) is approximately nine in 1,000 live births globally^[15,16]^ - in Africa it is 1.9 per 1,000 live births^[17]^ -. Survival rates vary by disease complexity, *e.g.*, the United States of America has a long-term survival (> 20 years) at approximately 95% for simple CHD, 90% for moderate complexity, and 80% for severe, complex CHD^[18]^. 3D printing can aid in ventricular assist device placement and optimizing function in complex CHD, as recently reported by Farooqi et al.^[19]^ and Saeed et al.^[20]^ in the United States of America and this can reduce the burden of this condition in Africa. By enabling the creation of customized prosthetics, models, and surgical guides, 3D printing can improve surgical outcomes, reduce complications, and enhance patient satisfaction making advanced surgical techniques more widely available across the continent^[21]^. This technology may be particularly beneficial in Africa, where access to specialized cardiothoracic care is often limited. Furthermore, 3D printing can facilitate pre-surgical planning, allowing surgeons to better understand complex anatomical structures and develop more effective surgical strategies. Additionally, it can enhance medical education and training, helping to address the shortage of skilled cardiothoracic surgeons in Africa^[21]^.

Currently, there is no published literature on the use of 3D printing technology in any African country within the domain of cardiothoracic surgery; hence, this is the first original article addressing this problem in the African continent. However, with a growing burden of cardiovascular diseases in Africa^[22]^, there seems to be a need for this technology as it has the potential to contribute to safer procedures, enhance surgical skills, and improve clinical outcomes^[11]^. This review aims to highlight the fundamentals of 3D printing, its relevance to current disease burdens in the African population, its current state and prospects in African cardiac care, and to know if there is a place for it in the field of cardiothoracic surgery in Africa.

## 3D PRINTING TECHNIQUES

3D printing is done by adding layers of synthetic materials to create a physical model from a digital 3D model reference. The creation of a 3D-printed model begins with an imaging exam, usually a CT, which stores data in digital imaging and communications in medicine (DICOM) format^[23]^ (Table 1).

**Table 1:** Different types of three-dimensional (3D) printing techniques, their advantages, drawbacks, and core techniques.

3D Printing Technique	Core Technique	Materials Used	Advantages	Disadvantages
Fused deposition modelling	Extrusion and deposition.	Thermoplastics such as polycarbonate, acrylonitrile butadiene styrene, polyphenylsulfone.	Easy to operate, low cost, fast, wide variety of usable thermoplastic materials for printing.	Relatively long print times, nozzle clogging, print resolution low compared to other types of printers.
Stereolithography	Laser scanning and ultraviolet-induced curing.	Photosensitive resins/polymer.	High resolution, detailed fabrication of internal structures, excellent print surface quality.	High cost related to the materials, printer, maintenance, relatively long print times, cytotoxicity.
Selective laser sintering (SLS)	Laser scanning and heat-induced sintering.	Chamber of powered materials including ceramics, metals, glass, and nylon.	Excellent surface quality and precision, does not require support structures, can print delicate structures, very large build volumes, can produce mechanically functional prints out of metals and ceramics.	Difficult to operate and calibrate; hence, it requires expert handling of the printer, high cost related to the materials, the printer, and the maintenance, requires post-production manual handling.
PolyJet	Inkjet-based photopolymerization	Photosensitive resins/polymers	High precision, medical-grade materials, very flexible models	Low strength and temperature resistance, high cost, low durability
Direct metal laser sintering (DMLS)	SLS/melting.	Stainless steel alloys, nickel-based alloys, aluminum alloys, titanium alloys.	DMLS can produce parts with improved mechanical properties, such as strength, toughness, and fatigue resistance. It allows for rapid production of parts, reducing lead times and increasing productivity.	DMLS machines require significant amounts of energy and maintenance, which can increase their operating costs. It involves the use of high-powered lasers and metal powders, which can pose safety risks if not handled properly.
Multi Jet Fusion (MJF)	Powder bed fusion.	Polyamide 12, polyamide 11, thermoplastic polyurethane.	MJF offers high resolution and detail, making it suitable for printing small features and intricate geometries.	MJF materials can be sensitive to humidity and temperature, which can affect the printing process and part quality.

**Fig. 1:**
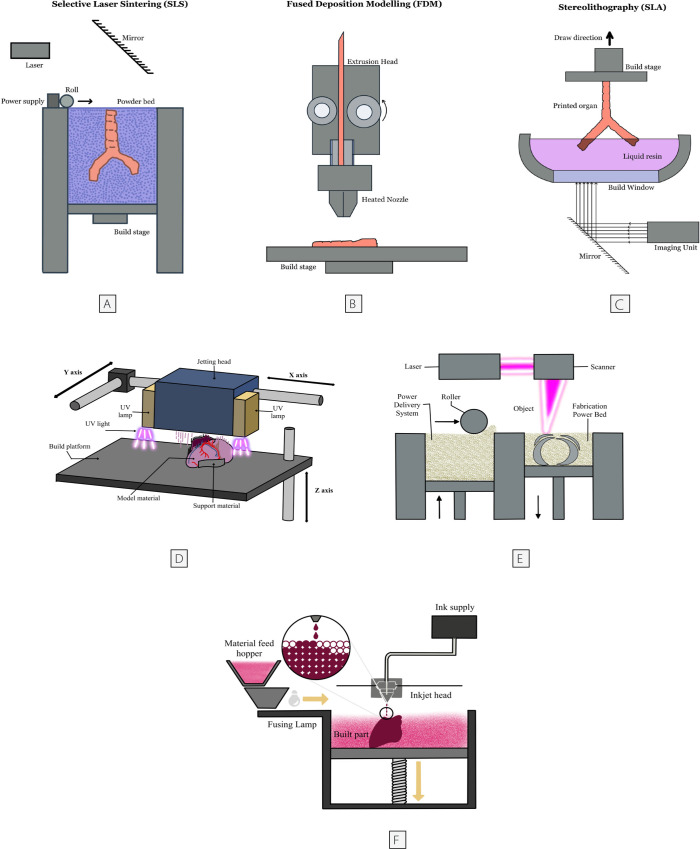
Commonly used three-dimensional techniques. (A) Selective laser sintering; (B) fused deposition modelling; (C) stereolithography; (D) PolyJet; (e) direct metal laser sintering; and (F) Multi Jet Fusion. UV=ultraviolet.

The main 3D printing techniques are fused deposition modelling (FDM), stereolithography (SLA), and selective laser sintering (SLS)^[24]^. FDM is a printing technique in which the printer heads are used to deposit melted lines of plastic onto a platform in layers^[25]^. This process is a quick prototyping technique. As plastic cools and hardens, a final model is created. FDM is not expensive and can use different types of plastic. It is the most frequently used technology for cardiovascular 3D printing^[26]^ (Figure 1B).

For SLA, it uses a photoreactive polymer as a base material. A light source is employed to solidify a layer of polymer liquid, and the printer keeps adding layers of thin liquid polymer until the whole model is solidified. Stroboscopic post-curing process may be employed to ensure that the photoreaction has been completed. This helps to boost the mechanical properties of the model^[27,28]^. However, this printing modality is expensive, and it is worth to note that the FDM and SLA techniques are more accessible than other printing techniques (Figure 1C).

In SLS, a laser fuses powder material layer by layer to create the desired object. Shot peening is then carried out to strengthen the outer layer. This type of printing is used to create nylon, ceramic, wax, metal, or composite parts. This printing technique is costly but produces very accurate models with smooth surfaces^[7,29]^ (Figure 1A).

PolyJet is another printing technique in which each layer of liquid polymer printed is solidified and cured via ultraviolet light. PolyJet can create smooth and accurate 3D models out of a huge array of materials for use as prototypes and parts, holding the capability to produce complex, multi-colored, and multi-material models with smoother and thinner walls. Due to the flexibility and complexity of the resulting models, PolyJet is also an ideal 3D printing approach for creating patient-specific cardiovascular models even though it is quite expensive (Figure 1D)^[30]^.

Direct metal laser sintering (DMLS) is a 3D metal printing method that uses a precise, high-wattage laser to micro-weld powdered metals to build objects out of almost any metal alloy^[31]^. During this process, a laser is slowly and steadily moved across the surface to sinter a very thin layer of spreading metal powders, which means that the particles inside the metal are fused together, although the metal is not heated enough to allow it to melt completely. In this way, DMLS gradually builds up a 3D object through a series of very thin layers, even porous metal components. One major limitation of this technique is that its machines are very expensive to maintain (Figure 1E).

Multi Jet Fusion is an innovative 3D printing technique that works similar to a binder jet technique in using a powder delivery system. However, the unique build style includes incorporation of a multi-agent inkjet system within the Powder Bed Fusion process^[32,33]^. The printing process involves the application of a thin layer of powder materials on the build plate followed by selective deposition of the fusing agent onto areas where the powder particles are intended to fuse and the addition of detailing agent at the contour of the patterns to create smooth surfaces. The powder layer on exposure to the infrared energy source allows the area of the fusion agent to fuse and forms the part. This technique is capable of fabricating parts with excellent dimensional precision and low porosity (Figure 1F)^[34]^.

## DEVELOPMENT OF CARDIOTHORACIC 3D-PRINTED MODELS

With advances in 3D printing technology, patient care can be improved. High-quality imaging data is needed to generate cardiac 3D-printed models. Preferably from MRI, transesophageal echocardiography, or CT imaging^[35]^, imaging datasets are exported into DICOM formats that are loaded into post-processing software^[36]^. Target anatomic geometry is identified and segmented from imaging datasets based on the threshold intensity of pixels in greyscale bidimensional image projections (coronal, axial, and sagittal). Segmentation masks are produced so that pixels with the same intensity range are grouped and assigned to be printed with single material. Then, segmentation masks are transformed into 3D digital models with rendering techniques^[36,37]^.

Next, these 3D digital models are saved as Standard Tessellation Language (STL) file format^[25]^, for further adjustment within the Computed aided Design software^[36]^. These adjustments may be texturing blended materials, color coding a region of interest, or including coupling components to evaluate the 3D-printed model within a flow loop^[38]^.

**Fig. 2:**
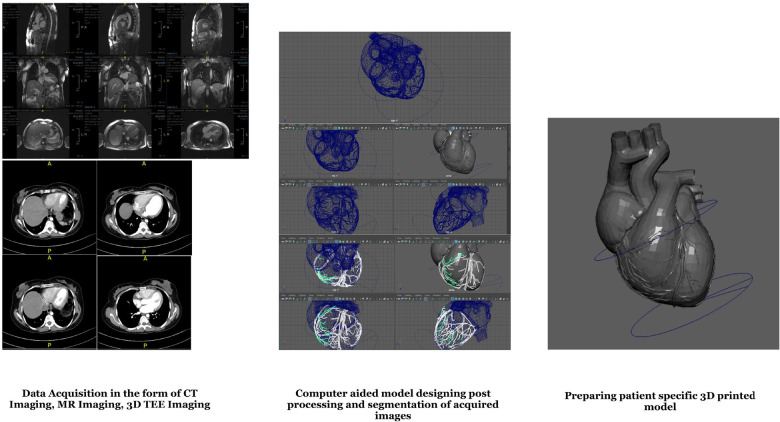
Workflow showing the processes involved in the development of a cardiac three-dimensional (3D) printed model. CT=computed tomography; MR=magnetic resonance; TEE=transesophageal echocardiogram.

Anatomic resolution can be further modified by combining datasets from various imaging perspectives. Moreover, segmentation can be improved via digital co-registration of DICOM data from complementary imaging modalities. The co-registration depends on pathoanatomy or discrete anatomy seen in both DICOM datasets, such as prosthetic material or focal calcification^[38,39]^ (Figure 2).

## HOW DOES 3D PRINTING FIT INTO THE NEEDS OF AFRICA? UNDERSTANDING THE NEEDS OF AFRICA CARDIOVASCULAR SURGERY

### Valvular Heart Disease

Valvular heart disease (VHD) in the Nigerian population is mainly of rheumatic origin and affects young to middle-aged individuals. The highest occurrence is seen in the mitral valve, followed by respectively aortic, tricuspid, and pulmonary heart valves. Ultimate treatment of rheumatic VHD is mainly surgical valve replacement or repair^[40]^.

3D-printed models of valve pathologies add value to surgical planning, helping in pre-interventional identification of complications and simulation of transcatheter aortic valve repair (TAVR) as well as transcatheter mitral valve repair (TMVR)^[41]^. Material flexibility is considered an important component in pre-surgical planning of procedures like TAVR for observation of the aortic root to select the optimum device for the patient^[42]^.

3D printing offers the prospect of incorporating and dynamically modelling patient-specific mitral valve replicas within mock circulatory systems (MCS). However, such a system is dependent upon the manufacture of mitral replicas exhibiting the biomechanical properties of human valves and accurate simulation of the haemodynamic milieu of mitral pathologies. In a study conducted by Mashari et al., they showed the feasibility of the haemodynamic evaluation of a 3D-printed mitral valve after TMVR within an MCS. Furthermore, the authors were able to derive pressure half time values from continuous wave Doppler and chamber pressures through pressure monitoring catheters. The major limitation of that study is the ability of the MCS to only generate sufficient pressures to allow diastolic modelling^[43]^.

### Aortic Disease

A review article focusing on the pattern and epidemiology of abdominal aortic aneurysms stated that the prevalence of the disease was up to 6.4% in the African population. Males were considered to be more affected as compared to females^[44]^.

An important application of 3D printing technology for aortic disease is vascular deformity mapping (VDM). With recent advances in 3D printing techniques, VDM of thoracic aortic aneurysms can produce models with variable properties like flexibility and color. Burris et al. have demonstrated the 3D nature of VDM on a patient's aortic anatomy for assessing aortic enlargement throughout the entire length and circumference of the vessel wall and also depicting aortic growth rate measurements as "heat map", however, these applications are still confined to research centers and are not yet integrated into clinical practice, even in highly specialized centres^[45]^. Dual material printing of patient-specific models can mimic anatomical and physiological properties of diseased valves on aortic stenosis. Hence, features like pressure gradient and flow acceleration can easily be simulated for study^[46]^. Previous attempts by Tang F. et al. showed that a customized aortic stent graft could be designed and manufactured with the assistance of 3D printing technology. These implants showed better geometric compliance and physical character in patients' bench tests and in-vivo implantation^[47]^.

3D printing has laid a significant impact on the evolution of surgical planning and treatment in complex coronary interventions^[48]^. Patient specific 3D-printed models have been available for medical education and clinical practice. 3D-printed coronary artery models have been utilized in surgical planning of coronary stenting as demonstrated by Sun Z et al.^[49,50]^. Previous studies by Gocer et al.^[51]^ mention the utilization of 3D-printed models to prepare optimum saphenous venous graft length and the anastomosis site aiding in reducing the operation time and improving patient outcomes.

For assisting heart surgeons in the aorta endovascular field, 3D printing has become an affordable reality and it is rapidly expanding its applications, both in surgical planning and in education and training of residents and students^[52-55]^. The use of 3D modeling for vascular simulations can provide training and education in either normal or complex anatomy for African cardiothoracic surgeons and residents doctors. In one study, it was reported that general surgery residents who prepared for endovascular abdominal aneurysm repairs using both 3D CT images and a 3D model performed better on a perioperative case-scenario questionnaire than residents who used only 3D CT images and no model^[56]^. Models also provide an ideal format for training, allowing trainees to engage with the procedure on their own time, also helping them to practice for rare pathologies that experienced surgeons may only encounter a limited number of times in their careers^[57]^. It can also provide haptic feedback which may be lacking in virtual reality simulations and has been shown to improve anatomical knowledge in students^[58,59]^. This would be beneficial to cardiothoracic surgeons, resident doctors, and medical students in the African continent.

### Coronary Artery Disease

A systematic review on prevalence of stroke and coronary artery disease (CAD) in Africa stated that the current rate of CAD is low; around 4.75% in South African population. However, exposure to cardiovascular disease risk factors by urban-rural residence, like poorer diets with higher caloric intake, greater sedentary behavior, and lower physical activity levels in urban compared to rural residents, leads to higher rates of obesity, diabetes, and hypertension in urban subjects and are likely to influence the development of CAD and stroke. Moreover, there is evidence that the exact epidemiology of CAD and stroke is higher as compared to health records owing to that fact that many cases remain undiagnosed^[60]^.

In CAD management, 3D-printed model-assisted benchtop flow simulations can help measure pressure changes at any locus in the model, ultimately mirroring the fractional flow reserve (FFR) at several simulated physiological blood flow conditions^[61]^.

Results have shown that precise and controlled flow simulations can be achieved and can facilitate detailed investigations of the flow changes due to coronary artery pathologies, ultimately helping during training scenarios for both interventional cardiologists and cardiac surgeons. Thus, it is possible to replicate FFR measurements that correlate with CT- and catheter-based measurements. Advantages of this benchtop system include the implementation of physiologically relevant waveforms, simulation of different flow rates using distal resistance adjustment, and simulation of wall compliance.

3D printing simulates geometric properties of the coronary vasculature and physiological conditions at both average resting and hyperemic coronary flow. It also enables features to change parameters such as compliance, heart rate, and peripheral resistance by controlling the valves, pump, or environmental temperature, enabling detailed study under vivid circumstances^[61]^.

The implantation of coronary artery stent is an effective way to relieve acute vascular occlusion. First, the location and stenosis degree of the diseased blood vessel are identified by coronary angiography. Then, the guide wire, catheter, balloon, and vascular stent are delivered along the artery to the stenotic lesions site of the coronary arteries through the puncture technique. The stent is fixed in the stenotic blood vessel through the pressure expansion of the balloon, so as to achieve the purpose of improving myocardial blood perfusion and maintaining vascular patency^[62]^.

### Congenital Heart Disease

CHD are one of the leading causes of childhood morbidity and mortality, affecting approximately 1% of the newborn African population^[63]^. Common CHDs in the Nigerian population are ventricular septal defects (VSD) (40.6%), patent ductus arteriosus (18.4%), atrial septal defects (ASD) (11.3%), and tetralogy of Fallot (11.8%)^[64]^. The burden of these diseases increases every five years. Hence, surgical management is always important to treat such cardiac conditions. Repair surgeries require a deep understanding of anatomy. Although 3D images can be obtained through current imaging modalities, their visualization is limited to a flat-screen presentation, hampering the complete understanding of the condition^[65]^.

Patient-specific 3D-printed models of pediatric hearts with congenital anomalies based on CT or MRI are excellent tools for understanding the complex cardiac anatomy of CHD^[65]^. These models can be used for preoperative planning and hands-on simulation training prior to the actual surgery. In ASD/VSD, 3D printing is helpful for intraoperative spatial navigation of occlusion devices and optimizing patch sizing for defect closure^[66]^. Kim et al. reported the use of 3D-printed models for occluder device sizing and selection of the approach to cross the defect in cases of a muscular VSD and a fenestrated ASD with a large atrial septal aneurysm^[67]^. Separate models of the intracardiac volumes (blood pool) and the myocardium plus vessel walls were also reported for a complicated case of a patient with transposition of great vessels, large VSD, ASD, and dextrocardia that had undergone a number of prior surgical interventions^[68]^.

## THORACIC SURGERY

**Fig. 3:**
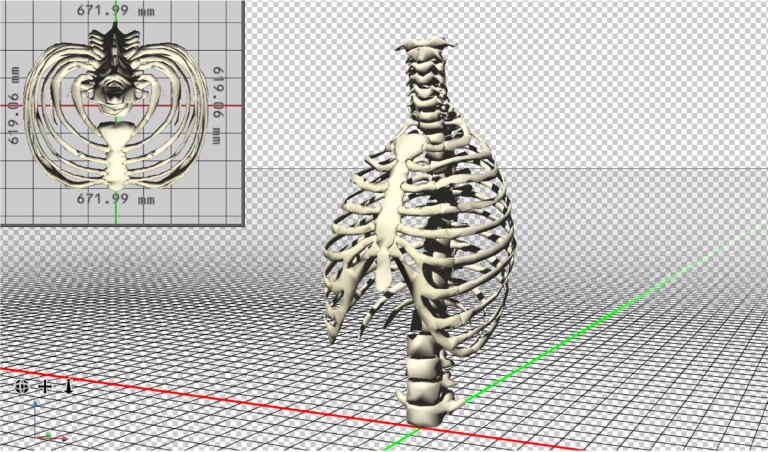
Image demonstrating computer-assisted three-dimensional (3D) printing model of the thoracic cage based on the patient details obtained from radiological images.

Thoracic deformities are not common in the African population^[66]^. Chest wall deformities are mainly presented as congenital chest wall deformities like pectus excavatum, pectus carinatum, Jeune's syndrome, tracheobronchomalacia, Poland syndrome, spinal deformities (kyphoscoliosis), pulmonary thromboembolism, mediastinitis, and thoracic tumors^[69]^ (Figure 3).

Regarding the minimally invasive repair of pectus excavatum, although there have been a lot of improvements to the original technique, decisions related to the number, location, and direction of implants are still made in the operating room with the patient under general anesthesia^[70]^. This results in a time-consuming procedure that may lead to a wrong selection of the length and configuration of the implants, thus requiring extensive re-bending, removal, and repetitive flipping of the bars. Also, bending the implant during surgery prompts the creation of scratches and notches that have been related to bleeding complications during the procedure or at bar removal^[70]^. However, the abovementioned setbacks may be addressed using 3D technology. On one hand, the 3D virtual reconstruction from a CT chest scan helps the surgical team to elaborate a detailed preoperative plan, including the number of implants required, their direction, and their entry points to the thoracic cavity. Computer programs have been developed to determine the precise length and shape of the prescribed implants, and STL files can be created for 3D printing of templates in materials such as polylactic acid that can be used for implant customization. On the other hand, 3D printing of real-size models of the chest wall or even the complete thoracic cage is being used for simulation, education, or tailored implant template manufacture as well^[70]^.

3D printing is useful in reconstructive thoracic surgery for preoperative planning and ambulatory template fitting in minimally invasive repair of pectus excavatum. Customized, pre-bent titanium implants (ribs and sternum) based on 3D-printed templates are suitable for rigid structure reconstruction demanding defect-specific precision in patient care. These implants provide minimal re-bending, reduced risk of implant flipping or removal after retrosternal passage, decreased postoperative complications, and improved functional capacity^[69,71-73]^. In 2019, Leng et al. reported using computer-aided designed 3D-printed cutting templates in four patients with pectus arcuatum with optimal results^[74]^. The wedge sternotomies and cutting templates were planned and designed virtually using 3D reconstructions of CT scans and then 3D-printed. Martinez et al. also published seven cases with complex chest wall deformities approached with a process using 3D technology between 2015 and 2020^[75]^. Diagnosis included isolated Poland syndrome (n = 1), pectus arcuatum (n = 2), Poland syndrome associated with pectus arcuatum (n = 3), and carinated deformity with complex sternal malformation (n = 1).

Alternatively, 3D-printed polyether-ether-ketone implants can be used in chest wall reconstructive surgery with post-surgical preservation of pulmonary function^[76-78]^. Recent studies have shown that a combination of 3D printing technology with a guide plate can effectively reduce bleeding, shorten the duration of operation, and increase the safety and accuracy of nail placement in the surgical management of thoracic spinal tuberculosis^[79]^. When combined with the framework of internal fixation technology, 3D printing has been advantageous in restoring the inherent shape of the thoracic cage, providing accurate and individualized treatment and reducing the operation difficulty in high complex rib fractures surgeries^[80]^. A study by Prakash et al. showed that 3D-printed prosthesis made from high-density polyethylene could also be used as an alternative implant option for large chest wall defect closure for eroded sternum after cure of mediastinitis^[81]^.

3D printing enables the manufacturing of vertebral bodies with a high degree of accuracy from non-contrast CT with minimal segmentation effort, provided that the high tissue-bone contrast and their utility in corrective surgery are rapidly being assessed^[82,83]^. A large-scale study in 126 adolescent idiopathic scoliosis patients used 3D-printed models of the entire spine to plan the corrective procedure, identifying complex or abnormal structures and simulating screw implantation. The outcomes of such spinal replacement with printed models were impressive. Patients had reduced rates of postoperative radiological complications, and hospital stay was also reduced^[84]^.

3D printing has also been exploited for the surgical management of tracheobronchomalacia, a condition characterized by the dynamic collapsing of the trachea and mediastinum bronchi. It offers a novel treatment by creating patient-specific, bioresorbable airway splinting. A recent ground-breaking application was described by Morrison et al. wherein 3D-printed bioresorbable tracheal splints were implanted in infants with life-threatening tracheomalacia. This is currently a clinically available solution for the surgical management of tracheobronchomalacia^[85]^. Similarly, Kaye et al. have demonstrated that a patient-specific tracheomalacia model can be prepared using 3D printing to reproduce the airway collapse, and this external splint can successfully treat the condition with promising results^[85]^. 3D-printed models of the airways are likely useful as training models. Tam et al. printed the tracheobronchial tree of a patient with advanced relapsing polychondritis complicated by tracheobronchial chondromalacia^[86]^.

3D printing also assists surgical planning for complex thoracic tumors. It can effectively help surgeons reduce operation time, reduce the risk of bleeding, and facilitate postoperative rehabilitation of patients by increasing surgical precision and reducing the risk of development of postoperative complications^[87]^. This is because the thoracic models allow pre-procedural simulation and rehearsal of the complicated procedures so that surgeons can be well-prepared for the actual procedure, thereby improving surgical outcomes including higher accuracy and increased postoperative functional scores. Posterior spinal fusion surgery can provide rigid intervertebral fixation. However, the angular misplacement of screws involves a high risk of neurovascular injury. 3D virtual planning and 3D-printed patient-specific drill guides appear safe and accurate for pedicle and lateral mass screw insertion in the cervical and upper-thoracic spine^[88]^.

## CURRENT REALITIES OF 3D PRINTING IN CARDIOTHORACIC SURGERY IN AFRICA

Although core cardiothoracic surgery practice is in its infancy in many countries in Africa, steps are being taken to institute the practice. In countries such as Ghana, Senegal, and Cote d’Ivoire, procedures such as open-heart surgery are done routinely. These practices had to contend with several challenges in terms of funding, staffing, and support. However, as the practices evolve, it is important to pursue global standards in operations and practice.

Consequent to the current paucity of cardiothoracic surgery practice in Africa, it may thus be deduced that 3D printing in Africa’s medical sector is also in its infancy. In the more economically advantaged countries, 3D printing is still experimental in most medical specialties, although reports have emerged of the process being used in various procedures. In Africa, there is very little published literature on the practice in use. African cardiothoracic societies and institutions have not released statements or proposed plans on how to incorporate the technology yet. Additionally, research output in the field of 3D printing as a whole is low. A recent analysis of Scopus showed that only 541 articles in the field of additive manufacturing and 3D printing were published between 2015 and 2021. Almost 80% of this research output is due to South Africa, which led the pile with 412 published papers^[89]^.

However, in South Africa, a recent partnership has led to the first manifestations of 3D printing in Africa’s medical practice. In 2020, Axial3D and MedTech3D partnered to design anatomical models for South African surgeons. Axial3D is a medical 3D printing company based in Belfast, Northern Ireland. MedTech3D is a local medical technology company that is working to provide 3D printing services to the medical and dental sectors in Africa. With the partnership, Axial3D will be able to provide 3D-printed models to Africa’s surgeons in various specialties, including cardiothoracic surgery, at affordable prices^[90]^. Even before this announcement, a team at Steve Biko Academic Hospital was able to perform middle ear operations with 3D-printed ossicles^[91]^.

In other specialties and applications of 3D printing in medicine, a few efforts have emerged as well. For example, in Bloemfontein, South Africa, there is the Medical Device Additive Manufacturing Demonstrator Project. Through this initiative, the company aims to produce medical devices, diagnostics, and pharmaceutical ingredients using additive manufacturing or 3D printing^[92]^. Ghana is another notable center where 3D printing’s potential in clinical practice is being explored, with the recent development of 3D prosthetic devices. In Uganda, the Comprehensive Rehabilitations Services hospital has worked with a consortium of Canadian organizations to trial 3D-printed prosthetic limbs for their patients^[93]^.

This indicates that Africa’s medical leaders and companies are willing to apply that technology to the medical sector. The potential benefits are myriad, especially within the African context. In Africa, issues such as shortages of organs and high costs of devices and prostheses can potentially be solved through 3D printing. This has already been indicated in various cases in Africa. A key example is the development of 3D-printed orthopedic device for hand and wrist injuries, through a collaboration between the William Davidson Institute and Ghanaian physical medicine and rehabilitation practitioners. The traditional design originally costs US$1,500 to make in Ghana, but the 3D-printed device costs only US$15^[94]^. The application of similar technologies to cardiothoracic surgery could help to significantly cut costs, improving access to cardiothoracic surgery in Africa. Potential uses of the technology, such as prosthetic manufacture, organ model bioprinting, and training, are desirable in Africa, as many of the issues faced in Europe and America are also present here.

## FUTURE PLANS AND RECOMMENDATIONS

The potential benefits of 3D printing to cardiac surgery practice in Africa should not be overlooked in stakeholders’ plans. However, as the practice begins to take its first steps across the continent, it is important to define priorities and how to solve challenges that will arise as 3D printing is introduced into cardiothoracic surgical practice.

### Funding and Infrastructure

3D printing requires specialized infrastructure, including devices for scanning patient organs and the 3D printers themselves. This technology is used to create complicated cardiac implants like heart valves, capable of mimicking intrinsic physicochemical and biomechanical properties of the cardiovascular system^[95]^. Currently, a medical-grade 3D printer can cost anywhere from US$20,000 to a few million for multi-material printers^[96]^. Considering the scale of these costs, it is important for proper financing approaches to be devised.

A solution is to look into public-private partnerships. Companies could take over the manufacturing process, partner with hospitals, and produce models on request, decreasing the need for hospital institutions to purchase 3D printers before offering associated services. Another solution may be to look for international grants. This may be helpful for instituting the initial stages of 3D printing in African cardiac surgery. Whichever approaches are put in place, government support will be essential. This will be expressed through funding as a primary approach, but also through other forms of support from its institutions.

### Establishing Cost-Effective 3D Printing Labs in Resource-Limited Regions with Sustainable Methods

Another recommendation which can help 3D printing in cardiothoracic surgery in Africa is the establishment of cost-effective 3D printing labs in resource-limited regions using sustainable methods. In Bengaluru, India, 3D template molds were created using locally available materials in a local start-up company, Osteo3D Inc^[97]^. The whole production cost was one-fifth of the total amount used in developed countries with an average estimate of about Indian rupees 20,000 (< $250 including all without any subsidy). The cost was less than a generic non molded titanium plate, and the production time is < 13 hours as the CT data can be uploaded online through a cloud-based software^[97]^. This method using 3D-printed molds is quick, straightforward, cosmetically accurate, and biomechanically stable. It also avoids direct contact of the implant to tissues caused by exothermic reaction and helps to create a smoother surface thereby reducing the risk of infection^[97]^. This can be adapted in the African continent

In Africa, Government mostly bears the cost of healthcare with Non-Governmental Organisations providing support once a while. Government agencies can play a key role in 3D printing in the field of cardiothoracic surgery by providing funding, supporting research and development initiatives, and ensuring regulatory oversight to guarantee the safety and efficacy of 3D-printed models. By actively supporting 3D printing in cardiothoracic surgery, governments can help alleviate the financial burdens of Africans associated with acquiring the necessary technology. Initiatives and grants from the government can play a great role in addressing the primary, secondary, and tertiary institutions financial constraints, enabling them to invest in 3D printing technology and training programs.

### Collaboration

International and inter-organizational collaboration will be essential in 3D printing in cardiac surgery in Africa. One recurring theme in the successful implementation of 3D printing in various projects in the African medical scene is international collaboration with firms, often from the West. The 3D-printed orthopedic device in Ghana, for example, was collaboratively developed by Ghanaians and a company in Michigan, United States of America^[94]^. Collaborations across the African continent will be essential in developing local capacity and sharing context-specific expertise. Collaboration will also be necessary for developing apropos solutions in African climes, such as devising materials, solving limited local demand, and other constraints. Hand in hand with this is the importance of research and development to ensure that the outcomes of trials and clinical cases are disseminated, facilitating evidence-based medicine.

### Access

Cardiothoracic surgery services, as a whole, are relatively expensive to the average African. This is compounded by the fact that out-of-pocket healthcare expenses are still common across Africa’s climes^[98,99]^. Open-heart surgeries in Africa can cost between US$4,000 and US$6,000, which happens to be a fraction of the cost of the same procedure in the Western world. However, with per capita gross national incomes in the order of US$2,000 or less, it is often difficult for citizens to afford cardiothoracic surgery^[100,101]^. Introducing 3D printing services can potentially bump costs, particularly in the initial phases, considering that operations will take time to become large-scale. Therefore, there is a need for action plans to make the service affordable while introducing it into cardiothoracic surgery practice in Africa. Health insurance is a valuable solution, provided it can cover the majority of the population.

### Feasibility

To make 3D printing feasible in resource-limited settings, such as Africa, conducting a thorough cost-benefit analysis will be essential. This will help to evaluate the return on investment and the long-term sustainability of integrating 3D printing technology into the domain of cardiothoracic surgery. This analysis can encompass a comprehensive assessment of the potential improvements in health outcomes, enhanced practical skills for cardiothoracic surgeons, and the overall impact on healthcare delivery in Africa. By systematically evaluating the costs against the anticipated benefits, African institutions can make informed decisions and allocate resources effectively.

## CONCLUSION

Even though 3D printing is not a new technology, it is a multi-versatile tool that has provided significant impact in the field of cardiothoracic surgery in different continents of the world already. However, it has been excluded in Africa due to lack of resources. 3D printing has been used for preoperative planning, surgical simulation, education, development of prosthetic implants, and creating models for communication with patients. Furthermore, with recent advances in biomaterials and 3D printing techniques, the technique has been used extensively for research.

Focusing on enhancing patient outcomes at minimal costs can greatly advance the adoption of 3D printing in cardiothoracic surgery across Africa. By emphasizing affordability, this technology can provide innovative solutions to cardiac and thoracic conditions in resource-limited settings. This approach would not only appeal to key stakeholders and potential investors but also position this subject matter in Africa for future global research. In turn, this can drive further innovations and investments, ultimately improving healthcare accessibility and quality across the continent.

Additionally, it is envisioned that this technology would have a significant impact on the African continent in the near future. Cardiothoracic surgeons in Africa would be able to plan surgeries, help residents and fellows to practice surgery, and better understand cardiac and thoracic conditions to improve the healthcare of Africans. A lot of innovations could be seen as cardiothoracic surgeons in Africa explore 3D printing in their research and clinical and educational endeavors.
